# Enhancement of host anti-mycobacterial immunity by green tea hot-water extract is mediated through miR-9-5p-enriched lung extracellular vesicles

**DOI:** 10.3389/fimmu.2026.1786162

**Published:** 2026-04-01

**Authors:** Jiayu Zhou, Liying Zhu, Yalin Wang, Yuan Wu, Min Yang, Wei Xu, Xiaosai Ma, Chonghai Qiu, Xiaoran Bian, Fangying Yi, Hongbo Shen, Feifei Wang

**Affiliations:** 1 Key Laboratory of Medical Molecular Virology (MOE/NHC/CAMS), Shanghai Institute of Infections Disease and Biosecurity, Department of Medical Microbiology and Parasitology, School of Basic Medical Sciences, Shanghai Medical College, Fudan University, Shanghai, China; 2 Shanghai Clinical Research Center for Infectious Disease (Tuberculosis), Shanghai Key Laboratory of Tuberculosis, Shanghai Pulmonary Hospital, Institute for Advanced Study, Tongji University School of Medicine, Shanghai, China; 3 Institute of Science and Technology, Fudan University, Shanghai, China; 4 State Key Laboratory of Genetic Engineering, Institute of Genetics, School of Life Science, Fudan University, Shanghai, China; 5 Shanghai Sci-Tech Inno Center for Infection and Immunity, Shanghai, China

**Keywords:** extracellular vesicles, green tea hot-water extract, gut-lung axis, miR-9-5p, *Mycobacteria tuberculosis*

## Abstract

Green tea, rich in bioactive compounds, is widely recognized for its immunomodulatory and anti-infection potential. In this study, we systematically evaluated the efficacy of green tea hot-water extract (GHWE) in modulating host antimycobacterial immunity. We first demonstrated that GHWE treatment enhanced the capacity of human peripheral blood mononuclear cells (PBMCs) to inhibit intracellular mycobacterial growth. This protective effect was further validated *in vivo*, where oral administration of GHWE significantly reduced the pulmonary bacterial burden in BCG-infected mice. Mechanistically, GHWE promoted the release of bactericidal extracellular vesicles (EVs) derived from bronchoalveolar lavage fluid (BALF). Specifically, miR-9-5p was markedly upregulated in BALF-EVs following GHWE administration and enhanced macrophage-mediated inhibition of intracellular mycobacteria by increasing reactive oxygen species (ROS) production. In addition, we observed that GHWE gavage also strengthened the antimicrobial activity of gut-derived EVs (GUT-EVs). Tracing experiments showed that GUT-EVs could travel through the circulatory system to the lungs, suggesting that they may mediate distal immune regulation along the gut-lung axis. Taken together, GHWE administration not only directly enhances the antimycobacterial capacity of host immune cells but also promotes cross-organ immune modulation by regulating EV-mediated communication between the gut and lung. This study provides new mechanistic evidence supporting the health-promoting functions of tea bioactive substances and highlights the importance of EV-mediated gut-lung interactions in dietary immunomodulation.

## 1 Introduction

Tuberculosis (TB), caused by *Mycobacterium tuberculosis* (*Mtb*) complex (1), is an infectious disease that remains one of the leading causes of death worldwide from a single pathogen. According to the World Health Organization, in 2024, an estimated 10.7 million people developed TB globally, with 1.23 million deaths. The continuous emergence of drug-resistant *Mtb* strains (2) and the complex immune evasion mechanisms of the pathogen pose significant challenges to TB control (3, 4). Therefore, developing novel adjunctive therapies or preventive strategies that enhance host anti-TB immunity by modulating cellular metabolism, inflammation, and immune responses holds substantial scientific and clinical significance.

In recent years, natural products and dietary components have attracted considerable attention due to their multiple bioactivities (5). Green tea, a widely consumed beverage globally, contains abundant bioactive compounds, with polyphenols representing the largest secondary metabolite family, accounting for approximately 35% of its dry weight (6). These diverse constituents, including the predominant polyphenol epigallocatechin gallate (EGCG), alongside key amino acids like theanine and alkaloids such as caffeine, have been demonstrated to exert significant anti-inflammatory (7), anticancer (8) and antimicrobial (9) effects by modulating cytokines, antioxidant systems, and immune-related signaling pathways. However, whether and how bioactive components of green tea can regulate host anti-tuberculosis immunity remains largely unexplored.

The gut and lung share similar mucosal immune architectures and are interconnected through systemic circulation and immune cell trafficking. The gut–lung axis refers to a complex communication network linking the gastrointestinal tract and the respiratory system via microbial, metabolic, and immunological signals. Emerging evidence highlights the role of the “gut-lung axis” in immune regulation (10, 11). Gut microbiota and their metabolites not only participate in local mucosal immunity but can also influence pulmonary inflammation and host defense through neural, metabolic, and immunological pathways (12). For instance, oral administration of *Lactobacillus plantarum* significantly restores host anti-tuberculosis immune responses and effectively reduces pulmonary bacterial burden in *Mtb* infected mice (13). *In vitro* studies further demonstrate that *Lactobacillus* species exert a pronounced inhibitory effect on mycobacterial growth, with their antimycobacterial activity likely attributable to secreted microbial metabolites (14). Beyond the microorganisms themselves, microbiota-derived or diet-associated metabolites also play important roles in regulating anti-tuberculosis immunity in the lung. For instance, palmitic acid treatment markedly reduces mycobacterial load in the lungs of *Mtb-*infected mice and alleviates histopathological damage, whereas palmitoleic acid suppresses tuberculosis antigen–specific TNF production and decreases TNF expression in both CD4^+^ and CD8^+^ T cells (15). Moreover, during *Mtb* infection, parenteral vaccination has been shown to induce gut dysbiosis-related metabolic changes and promote the generation of lung-resident memory macrophages and trained immunity (16). Collectively, these findings underscore the close and functionally significant involvement of the gut–lung axis in shaping host immune responses during *Mtb* infection.

Extracellular vesicles (EVs), present in blood, respiratory secretions, and intestinal luminal fluids, carry miRNAs, mRNAs, proteins, and lipids, and mediate intercellular communication by modulating gene expression and cellular functions (17). As critical biomarkers, EVs secreted by various cells are closely associated with pulmonary homeostasis and diseases (18, 19). Particularly during *Mtb* infection, miRNAs exert a profound impact on the survival and clearance of intracellular pathogens by regulating macrophage phagocytosis, autophagy, and cellular metabolism. Studies have shown that *Mtb* infection alters the quantity and composition of miRNAs in macrophage-derived EVs, and significant differences exist in the serum EV miRNA profiles between TB patients and healthy individuals (20).Infected macrophages are capable of transferring specific microRNAs (miRNAs) to target cells via EVs, thereby playing a pivotal role in driving the progression of infectious diseases and modulating host anti-infective immune responses. Notably, miRNAs such as miR-21, miR-27a, and miR-155 have been extensively documented as key regulators closely associated with the activation status and immune functional profiles of macrophages (21). Similarly, the expression of miR-107 in mouse plasma EVs is significantly upregulated, which inhibits intracellular mycobacterial growth by modulating autophagy-related signaling pathways (22). Conversely, *Mtb* can manipulate host miRNAs for immune evasion; for example, EVs released by *Mtb*-infected macrophages are enriched with miR-18a, which interferes with autophagy signaling and weakens the host’s ability to clear mycobacteria (13). Evidently, EV-mediated miRNAs exert dual regulatory roles—both immunoprotective and immunosuppressive—during TB infection. However, their overall profile changes and systemic functions under specific dietary interventions remain to be fully elucidated. Moreover, the green tea polyphenol EGCG can significantly alter the composition of cell-secreted EVs: it reshapes the proteomic profile of intestinal epithelial cell-derived EVs (23) and regulates miRNA content in tumor cell-derived EVs, thereby influencing macrophage phenotype and immune function (24). These findings provide important theoretical support for the synergistic regulatory mechanisms linking dietary bioactive compounds, EVs, and immune modulation.

To address this, the present study employed green tea hot-water extract (GHWE) as an intervention, using both *in vivo* gavage and *in vitro* cell culture models to systematically assess its effects on gut-lung axis mediated immune function. We found that: (1) Oral GHWE administration could enhance the anti-TB effects of host bronchoalveolar lavage fluid-derived EVs (BALF-EVs); (2) these EVs were highly enriched in miR-9-5p, which subsequently restricted intracellular *Mtb* growth in macrophages; and (3) miR-9-5p promoted macrophage reactive oxygen species (ROS) production, thereby enhancing antibacterial responses. Overall, this study reveals how green tea compounds bolster anti-TB immunity by influencing EVs and their regulatory miRNAs along the gut-lung axis. These findings support the use of dietary components in TB adjunctive therapy and pave the way for developing host-directed immunomodulatory strategies.

## 2 Materials and methods

### 2.1 Human subjects

This study was approved by the Institutional Review Board for human subject research and the Institutional Biosafety Committee of Shanghai Pulmonary Hospital (SPH), Tongji University (approval number: K22-143Z). All participants were adults and provided written informed consent prior to enrollment. All procedures were conducted in strict accordance with the protocol approved by the Ethics Committee of Shanghai Pulmonary Hospital, Tongji University.

### 2.2 Peripheral blood mononuclear cell isolation and cell culture

Peripheral blood samples were collected from healthy donors at Shanghai Pulmonary Hospital. Peripheral blood mononuclear cells (PBMCs) were isolated using Ficoll-Paque density gradient centrifugation as previously described (25). Briefly, peripheral blood was centrifuged, and the buffy coat layer was collected and gently mixed with culture medium. The cell suspension was carefully layered onto Ficoll-Paque and centrifuged to allow density gradient separation. The mononuclear cell layer was then carefully aspirated, transferred to a new tube, and washed. Following red blood cell lysis and additional centrifugation and washing steps, purified PBMCs were obtained. PBMCs were cultured at 37°C in medium supplemented with 10% fetal bovine serum (FBS, Gibco) and 100 U/mL penicillin-streptomycin.

### 2.3 Animal experiments

The animal study protocol was approved by the Institutional Animal Care and Use Committee of Fudan University (protocol code A20250022).

#### 2.3.1 Oral administration of GHWE in mice

Green tea hot-water extract (GHWE) was prepared by extracting 2 g of Lipton green tea leaves with boiling water at a 1:45 (w/v) ratio for 45 min. The extract was cooled and filtered for subsequent experiments and chemical characterization. Liquid chromatography-mass spectrometry (LC–MS) was subsequently employed to characterize GHWE and to analyze its major chemical constituents, which are summarized in **Supplementary Table 1**.

Specific pathogen-free (SPF) C57BL/6J mice (6–8 weeks old) were randomly assigned to two groups (n = 8 per group): the GHWE intervention group and the phosphate-buffered saline (PBS) control group. Mice were administered 200 μL of GHWE or PBS by oral gavage once daily, 5 days per week for 2 consecutive weeks, followed by sample collection. Concerning the GHWE gavage dose, the selected dose of 400 mg/kg/day in mice was based on previous studies evaluating the immunomodulatory and antimicrobial effects of green tea extracts (26, 27). No signs of adverse effects or body weight loss were observed throughout the intervention period.

#### 2.3.2 Mouse sample collection

Mice were anesthetized via intraperitoneal injection of a 20 mg/mL tribromoethanol solution (Nanjing Aibei Biotechnology Co., Ltd., Nanjing, China) at a dose of 200 mg/kg. Upon reaching a surgical plane of anesthesia, confirmed by the loss of the pedal withdrawal reflex, the mice were euthanized by cervical dislocation. Subsequently, the thoracic cavity was opened to expose the lungs and trachea for sample collection. A needle was inserted into the trachea, and 2 mL of PBS was gently perfused to collect BALF. Samples from each group were pooled for subsequent processing.

Gut extracellular vesicles (GUT-EVs) were isolated from mouse intestinal epithelial cells, and the epithelial cell isolation procedure was performed as follows. Briefly, a segment of the small intestine extending from the distal duodenum to approximately 5 cm proximal to the colon was excised, and mesenteric adipose tissue was carefully removed. The intestine was immediately placed in ice-cold PBS, and the lumen was thoroughly flushed in both directions with pre-cooled PBS to completely remove luminal contents and fecal material. Residual adipose tissue and Peyer’s patches were removed, after which the intestine was longitudinally opened, cut into small fragments, and washed three times with PBS. The tissue was incubated in PBS containing 10 mmol/L dithiothreitol (DTT) for 5 min to remove mucus, followed by incubation in PBS containing 8 mmol/L EDTA for 30 min to dissociate intestinal epithelial cells. After vigorous shaking in fresh PBS, the suspension was centrifuged at 300 g for 10 min to remove tissue debris. The resulting supernatant, enriched in intestinal epithelial cells, was collected and used for EV isolation (28, 29).

### 2.4 Cell culture

MLE12, RAW264.7, and THP-1 cell lines were obtained from the Cell Bank of the Chinese Academy of Sciences (Shanghai, China) and maintained in Dulbecco’s modified Eagle’s medium (DMEM) or RPMI 1640 medium, as appropriate. All media were supplemented with 10% FBS and 100 U/mL penicillin-streptomycin. Cells were cultured at 37°C in a humidified incubator with 5% CO_2_.

### 2.5 Mycobacterial culture and cell infection

*Mycobacterium bovis* BCG (ATCC 35733) and *Mycobacterium smegmatis* [ATCC 00084/mc² 155] were cultured at 37°C in Middlebrook 7H9 broth supplemented with 10% OADC, 0.05% Tween-80 and 0.2% glycerol (30, 31).

RAW264.7 cells were seeded in 96-well plates and infected with BCG at a multiplicity of infection (MOI) of 5. After 4 h of infection, cells were washed and treated with 5 µg/200µl of EVs to assess intracellular bacterial control. THP-1 cells were infected with BCG (MOI = 1) and incubated under the same conditions for 3 days to evaluate intracellular bacterial control. At the end of incubation, cells were lysed or collected by centrifugation, plated on 7H10 agar supplemented with OADC, and incubated for 2–3 weeks before enumerating colony-forming units (CFUs).

### 2.6 Cell transfection

THP-1 or RAW264.7 cells were cultured to an appropriate density under antibiotic-free conditions prior to transfection. miRNA mimics, inhibitors, and their corresponding negative controls were diluted in serum-free Opti-MEM and mixed with Lipofectamine™ 2000 (Thermo Fisher Scientific) diluted in the same medium. After incubation at room temperature to allow complex formation, the transfection mixtures were added to the cells. Following a 4–6 h transfection period, the medium was replaced with complete medium supplemented with 10% exosome-depleted FBS. For overexpression groups used for EV collection, cells were further cultured for 48 h, and culture supernatants were harvested for EV isolation and subsequent experiments.

### 2.7 Reactive oxygen species assay

THP-1 cells (6 × 10^4^ cells per well) were seeded in 96-well plates and transfected with miR-9-5p mimics or a corresponding mimic negative control (NC), miR-9-5p inhibitors or the corresponding inhibitor NC. After 48 h of transfection, cells were processed using a ROS detection kit (Solarbio) according to the manufacturer’s instructions. Briefly, cells were loaded with the fluorescent probe DCFH-DA (10 μM) and co-incubated for 30 min at 37°C in the dark. Fluorescence intensity, directly reflecting intracellular ROS levels, was subsequently measured using a microplate reader.

### 2.8 Extracellular vesicle isolation

Following epithelial cell dissociation, the collected intestinal epithelial cells were resuspended in ice-cold PBS and incubated on ice for 30 min to allow extracellular vesicle release. The cell suspension was then centrifuged at 3000 g for 20 min to remove intact cells, followed by spin at 12000 g for 30 min to eliminate cell debris and large vesicles. The resulting supernatant was filtered with a 0.22 μm syringe filter (Millipore). Small extracellular vesicles were subsequently pelleted by ultracentrifugation at 110000 g for 70 min at 4°C. After removal of the supernatant, the pellet was resuspended in ice-cold PBS and subjected to a second ultracentrifugation at 110000 g for 70 min at 4°C for washing. Finally, the supernatant was discarded, and the extracellular vesicle pellet was resuspended in an appropriate volume of ice-cold PBS for downstream analyses. For GUT-EVs experiments, samples were pooled from 8 mice per biological replicate.

BALF samples were subjected to sequential centrifugation for pre-clearance. Briefly, samples were centrifuged at 300 g for 10 min to remove cells, followed by 3000 g for 20 min to eliminate cell debris, and 12000 g for 30 min to remove large vesicles. The resulting supernatant was then filtered through a 0.8 μm syringe filter (Millipore). Extracellular vesicles were isolated using the exoRNeasy Exosome Isolation Kit (QIAGEN) according to the manufacturer’s instructions. After using a bicinchoninic acid (BCA) protein assay kit (Beyotime), extracellular vesicles were stored at -80 °C.

Due to the limited yield of EVs from individual mice, a sample pooling strategy was employed. For GUT-EVs, each biological replicate consisted of pooled samples from 2 individual mice. For BALF-EVs, each biological replicate was derived from a pool of 8 mice.

### 2.9 Transmission electron microscopy

A 2 μL aliquot of the BALF-EVs sample was applied onto a carbon-coated copper grid and allowed to adsorb for 10 min at room temperature. Excess liquid at the grid edge was gently blotted with filter paper. The grid was then negatively stained with 2% phosphotungstic acid for 5 min at room temperature. After blotting and air-drying, the negatively stained samples were examined using a transmission electron microscope, and images were captured to document EV morphology.

### 2.10 Nanoparticle tracking analysis

BALF-EVs were diluted in sterile PBS to a final concentration of 10^6–^10^9^ particles/mL. A 2 μL aliquot of the diluted sample was then loaded for nanoscale particle size analysis.

### 2.11 Western blot analysis

Protein concentrations were determined using a BCA protein quantification kit (Beyotime). Equal amounts of protein were mixed with 5× SDS loading buffer, separated on 10-12% SDS-PAGE gels and transferred onto 0.22 μm PVDF membranes (Millipore). Membranes were blocked at room temperature using a rapid blocking buffer (Beyotime) and subsequently incubated with primary antibodies overnight at 4°C. Primary antibodies included anti-CD63 (1:1000, Abcam) and anti-TSG101 (1:1000, Abcam). After washing, membranes were incubated with HRP-conjugated secondary antibodies (1:5000, Abcam) at room temperature for 1 h. Protein bands were visualized using an ECL detection reagent (Omni-ECL™ Chemiluminescence Kit, Epizyme) and imaged using a ChemiDoc imaging system (Bio-Rad).

### 2.12 *In vivo* fluorescence imaging in mice

An appropriate volume of DiR stock solution was added to EVs to achieve a final concentration of 15 μM, followed by incubation at room temperature for 30 min. Unbound dye was removed by ultracentrifugation at 120000 g for 90 min at 4°C. The EV pellet was washed once with PBS under the same ultracentrifugation conditions and then resuspended in an appropriate volume of PBS. DiR-labeled GUT-EVs (containing 2 μg DiR and 30 μg EV protein) were injected into mice via the tail vein (32, 33). Fluorescent images and organ distribution were captured at 2, 6, 24, and 48 h post-injection using an *in vivo* small-animal fluorescence imaging system.

### 2.13 EVs uptake assay

According to the manufacturer’s instructions (Sigma), GUT-EVs were resuspended in 500 μL Diluent C to prepare a 2× suspension. Briefly, 2 μL of PKH26 dye was mixed with 500 μL Diluent C and immediately combined with the EV suspension. After gentle mixing, the reaction was incubated at room temperature for 2–5 min, followed by addition of stop solution and incubation for 1 min. Unbound dye was removed by ultracentrifugation at 120000 g for 70 min at 4°C. The pellet was washed once with PBS and resuspended for subsequent experiments.

RAW264.7 cells were seeded at a density of 1 × 10^5^ cells per dish in confocal culture dishes and incubated overnight. After washing times with PBS, PKH26-labeled GUT-EVs (50 μg/mL) were added and incubated at 37°C for 12 h. Cells were then washed three times with PBS, fixed with 4% paraformaldehyde for 30 min, and counterstained with DAPI (1 μg/mL) for 10 min in the dark. After washing, an antifade mounting medium was applied, and images were acquired using a laser scanning confocal microscope.

### 2.14 RNA sequencing and analysis

Total RNA from EV samples was quantified with Qubit and assessed for integrity using an Agilent 2100 Bioanalyzer; only high-quality samples were used for library construction. For total RNA libraries, rRNA was depleted, cDNA synthesized with random primers and a template-switching sequence, followed by PCR, rRNA removal, and purification to generate strand-specific libraries. For small RNA libraries, adapters were ligated, followed by reverse transcription, PCR, and PAGE purification of ~140–160 bp miRNA libraries. Libraries were quantified, quality-checked, and sequenced on an Illumina platform.

Raw reads were processed for quality control and aligned to the mouse reference genome (GRCm39). Differential expression analysis was performed using DESeq2 or edgeR, with significance thresholds set at |log_2_FC| ≥ 1 and *p* < 0.05. Detailed data regarding the miRNA sequencing are presented in **Supplementary Table 2**.

### 2.15 RNA extraction and RT-qPCR

Total RNA from cells was extracted using TRIzol method. Briefly, cells were lysed in TRIzol, subjected to chloroform extraction and isopropanol precipitation, and the resulting RNA pellet was washed with 75% ethanol and dissolved in DEPC-treated water. RNA concentration and purity were measured using a NanoDrop spectrophotometer. RNA from EVs was extracted using the exoRNeasy Midi Kit (QIAGEN) according to the manufacturer’s instructions.

The total protein concentration of isolated EVs from independent biological replicates was determined using a BCA assay. Total RNA was subsequently extracted from standardized equal amounts of EV protein equivalents across all groups. miRNA reverse transcription was carried out using the miRcute Enhanced miRNA cDNA First-Strand Synthesis Kit (Tiangen). Quantitative reverse transcription PCR (RT-qPCR) was performed with the miRcute Enhanced miRNA qPCR Detection Kit (SYBR Green, Tiangen). Each 20 μL reaction contained 2× SYBR qPCR Mix, miRNA-specific primers, and template cDNA. The amplification protocol consisted of an initial denaturation at 95 °C for 15 min, followed by 40 cycles of 94 °C for 20 s and 60 °C for 34 s. U6 snRNA was used as the internal control, and relative expression levels were calculated using the 2^-^ΔΔCt method.

### 2.16 Data analysis

Statistical analyses were performed using GraphPad Prism 10.0. All data are presented as mean ± standard deviation (SD). A p-value < 0.05 was considered statistically significant. Comparisons between two groups were conducted using unpaired t-tests, while differences among three or more groups were analyzed using one-way ANOVA.

## 3 Results

### 3.1 Oral administration of GHWE enhances host resistance to mycobacterial infection and increases antimicrobial ability of EVs isolated from lung bronchoalveolar lavage fluid

**Figure 1:**
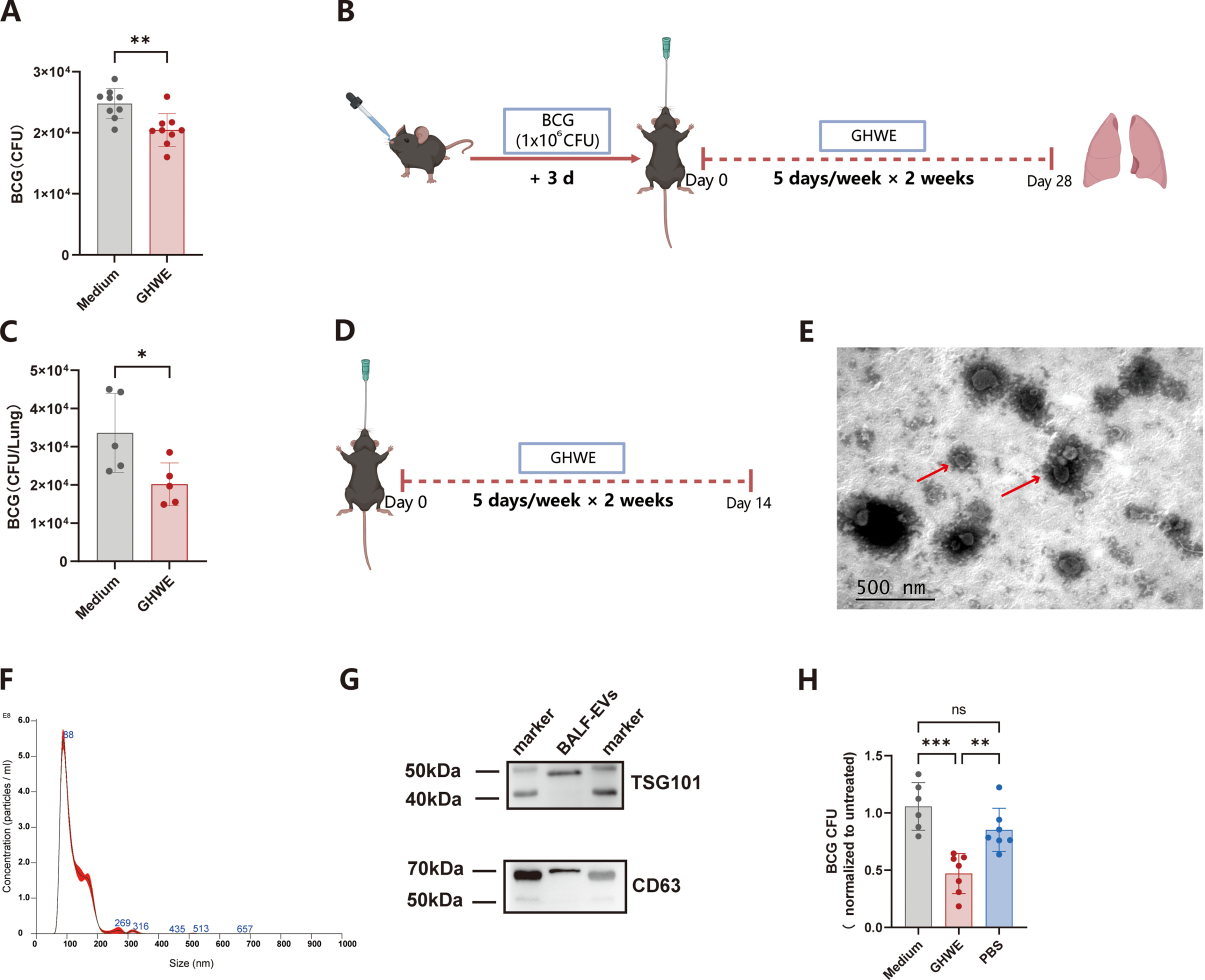
Oral GHWE administration enhances anti-tuberculosis immunity in mice and mediates antimicrobial activity via BALF-EVs. **(A)** PBMCs were stimulated with 100-fold dilution of GHWE for 3 days and subsequently co-cultured with BCG-infected THP-1 cells for an additional 3 days. Cells were then lysed, serially diluted, plated on 7H10 agar, and BCG CFU were enumerated. **(B)** Schematic representation of the mice experiments. Mice were infected intranasally with BCG and administered GHWE by oral gavage (5 days per week for 4 weeks). On day 28, mice were euthanized and lung tissues were collected for bacteria burden quantification. **(C)** Lung tissues were homogenized, serially diluted, plated, and BCG CFU were quantified. **(D)** Mice received GHWE by oral gavage (5 days per week for 2 weeks), and BALF was collected on day 14. **(E)** TEM of BALF-EVs, showing the characteristic cup-shaped vesicular morphology (scale bar, 500 nm). **(F)** NTA showing the size distribution and concentration of extracellular vesicles. **(G)** Western blot analysis of EV marker proteins TSG101 and CD63. The membrane regions corresponding to 40–50 kDa and 50–75 kDa were exposed and analyzed for TSG101 and CD63, respectively. **(H)** RAW264.7 cells infected with BCG were treated with BALF-EVs (5 μg/200μl) isolated from PBS- or GHWE-administrated mice for 3 days. BCG CFU were quantified thereafter. n=7 independent biological replicates per group (each replicate pooled from 8 mice). Results are expressed as mean ± SD. *p < 0.05, **p < 0.01, ***p < 0.001. ns p > 0.05. Statistical significance was determined using Student’s t-test(A,C) or one-way ANOVA(H). Data represent 3 independent experiments.

To evaluate the functional effects of green tea bioactive components in host defense against mycobacterial infection, we first prepared GHWE and stimulated human PBMCs for 3 days. Treated PBMCs were then co-cultured with BCG-infected THP-1 cells for an additional 3 days. Cell lysates were collected, serially diluted, and plated on 7H10 agar for CFU enumeration. The GHWE-treated group exhibited an average BCG burden of approximately 2×10^4^ CFU/well, significantly lower than the 2.5×10^4^ CFU observed in the untreated control, indicating that GHWE enhances the ability of human immune cells to restrict intracellular mycobacterial growth (**Figure 1A**). We next employed mycobacteria infected mice model to further assess the *in vivo* role of GHWE in modulating host antimycobacterial immunity. Mice were intranasally infected with BCG and subsequently administered GHWE(400 mg/kg/day) by oral gavage for 2 weeks (**Figure 1B**). To ensure physiological relevance, this dosage was selected based on body surface area normalization. It translates to a human-equivalent dose of approximately 1.95 g/day for a 60-kg adult, which is comparable to the polyphenol intake achievable by consuming roughly 4 cups of standard green tea daily—a level well within the safe and realistic range for healthy adults. Four weeks post-infection, lungs were harvested and homogenized for CFU determination. GHWE-treated mice displayed lung BCG loads of approximately 2×10^4^ CFU/lung, significantly reduced compared with 3.3×10^4^ CFU/lung in control mice (**Figure 1C**), demonstrating that GHWE effectively enhances host resistance to BCG infection.

Given the multifunctional roles of EVs in immune responses, we next examined whether EVs in the lung microenvironment contributed to the enhanced antimycobacterial effects. Mice were subjected to oral GHWE administration (5 days/week for 2 weeks), and BALF was collected on day 14 for EV isolation using the standardized exoRNeasy kit (**Figure 1D**). Transmission electron microscopy revealed round, membrane-bound vesicles with a characteristic cup-shaped morphology of BALF-EVs (**Figure 1E**), while nanoparticle tracking analysis showed a predominant particle size distribution within the expected range of exosomes, with a peak diameter of approximately 88 nm and the majority of particles ranging between 70–200 nm (**Figure 1F**). The particle concentration was determined to be 3.16 × 10^10^ particles/mL, which falls within the commonly reported concentration range for EV preparations (10^7^–10^12^ particles/mL), indicating efficient vesicle enrichment. Western blot analysis further confirmed the enrichment of canonical EV markers, CD63 and TSG101, supporting the successful isolation and relative purity of the vesicle preparations (**Figure 1G**). Functionally, BALF-EVs from PBS-treated mice presented a modest reduction in intracellular BCG growth in macrophages (relative CFU index= 0.85), although the difference from the medium control was not statistically significant (**Figure 1H**). Interestingly, BALF-EVs from GHWE-treated mice markedly suppressed intracellular BCG growth, reducing the relative CFU index to 0.47, significantly lower than both the medium and PBS groups (**Figure 1H**). Notably, the mean CFU value of the PBS-EV group was approximately 1.8-fold higher than that of the GHWE-EV group, indicating a substantial enhancement of EV-mediated antimycobacterial activity following GHWE administration.

Collectively, these results demonstrate that GHWE administration promotes the antimycobacterial capacity of lung-derived EVs, thereby contributing to improved host resistance to mycobacterial infection.

### 3.2 BALF-derived EVs from GHWE-treated mice exhibit elevated levels of miR-9-5p

**Figure 2:**
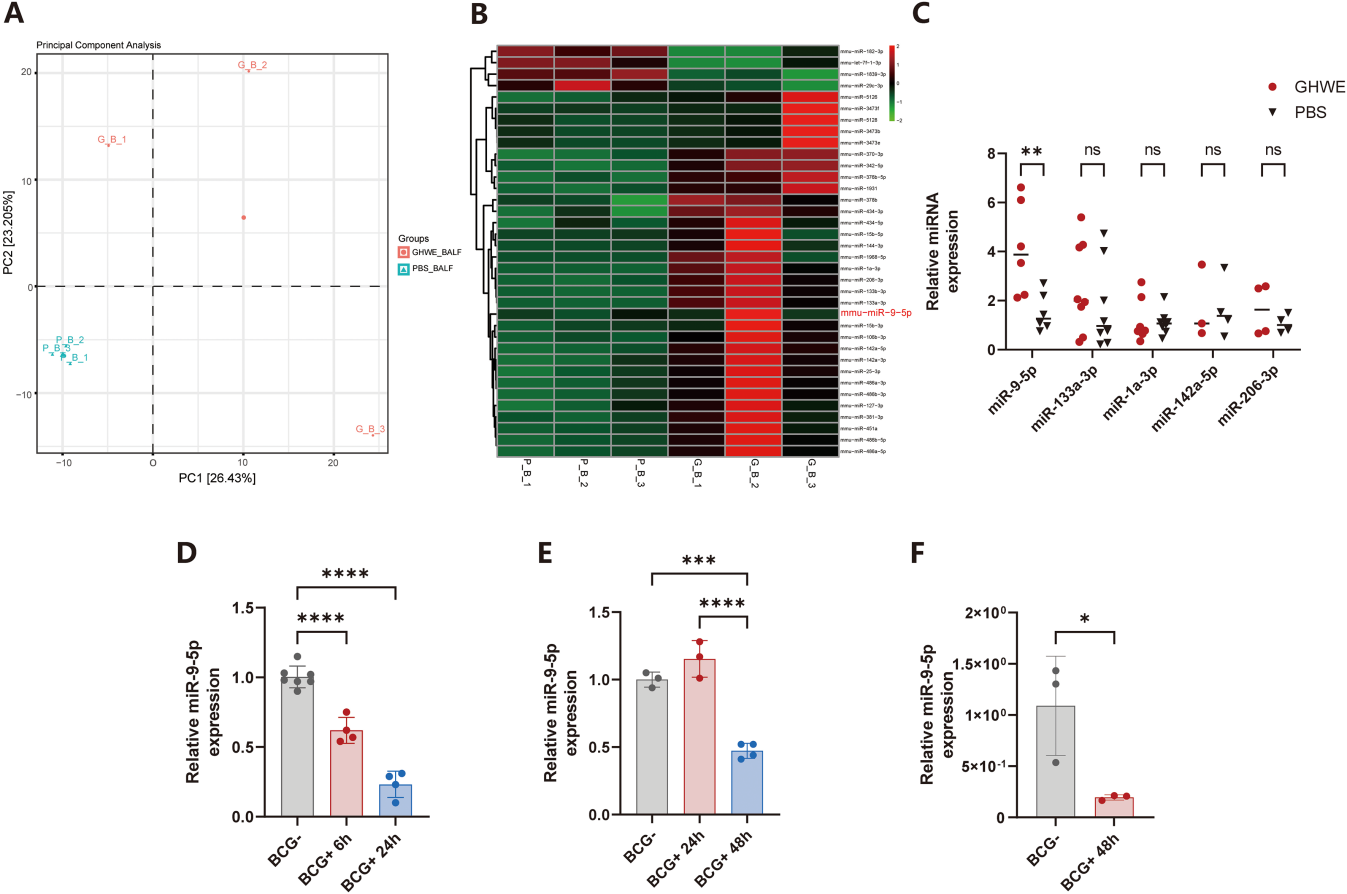
GHWE administration induces miRNAs composition alterations in BALF-EVs and identifies miR-9-5p as a key miRNA associated with anti-mycobacterial infection. **(A)** miRNA sequencing of BALF-EVs followed by principal component analysis (PCA). **(B)** Heatmap showing the top 50 differentially expressed miRNAs between BALF-EVs isolated from GHWE treated mice and control mice. **(C)** RT-qPCR detection of selected miRNAs in BALF-EVs from GHWE-treated mice and controls. Expression levels were normalized to equal EV protein input with U6 as an internal reference. **(D)** RAW264.7 cells were infected with BCG (MOI = 5), and total microRNA was extracted at 6 hours(h) and 24 h post-infection for RT-qPCR analysis of miRNA expression. **(E)** MLE-12 cells were infected with BCG (MOI = 1), and total microRNA was extracted at 24 h and 48 h for RT-qPCR analysis of miR-9-5p expression. **(F)** RAW264.7 cells were infected with BCG (MOI = 5), and total microRNA was isolated from extracellular vesicles at 48 h post-infection for RT-qPCR analysis of miR-9-5p expression. Results are expressed as mean ± SD. ns p > 0.05, *p < 0.05, **p < 0.01, ***p < 0.001, ****p < 0.0001. ns p > 0.05. Statistical significance was determined using Student’s t-test **(C, F)** or one-way ANOVA **(D, E)**. Data represent 3 independent experiments.

To investigate the mechanism by which GHWE enhances anti-mycobacterial immunity through BALF-EVs, and given previous evidence that EVs carry functional miRNAs, we performed whole-transcriptome sequencing to profile miRNA expression in BALF-EVs isolated from PBS- and GHWE-treated mice. Principal component analysis (PCA) revealed a clear separation between the two groups, indicating that GHWE markedly alters the miRNA composition of BALF-EVs (**Figure 2A**). Differential expression analysis identified 36 dysregulated miRNAs, among which 32 were significantly upregulated and 4 were downregulated in the GHWE group (|log_2_FC| > 1, *p* < 0.05). A clustered heatmap of the top 50 miRNAs further illustrated distinct expression patterns between the two groups (**Figure 2B**). These findings suggest that GHWE reshapes the miRNA landscape of BALF-EVs, implicating specific EV-associated miRNAs in regulating anti-tuberculosis immune responses.

To validate the sequencing results, we collected additional samples and performed RT-qPCR on selected differentially expressed miRNAs. Consistent with the sequencing data, miR-9-5p was markedly enriched in BALF-EVs from GHWE-treated mice (**Figure 2C**). miR-9-5p has previously been reported to participate in inflammatory regulation and immune cell activation (34), suggesting that it may serve as a critical mediator of the GHWE-enhanced, EV-mediated anti-mycobacterial activity.

### 3.3 Mycobacterial infection markedly suppresses miR-9-5p expression in BALF-derived EVs

To further elucidate the role of miR-9-5p in host defense against mycobacterial infection, we first examined its dynamic expression during BCG infection. RAW264.7 macrophages were infected with BCG, and miRNA levels were quantified by RT-qPCR. At 6 h post-infection, the relative expression level of miR-9-5p decreased to approximately 0.6(relative to the uninfected control set to1 was normalized to 1), representing a 40% reduction (**Figure 2D**). By 24 h, the relative expression of miR-9-5p further declined to 0.23, corresponding to only 20% of the control levels (**Figure 2D**). These results demonstrate that miR-9-5p is significantly downregulated following BCG infection, with a pronounced time-dependent suppression.

A similar pattern was observed in the murine lung epithelial cell line MLE-12. miR-9-5p levels showed no significant change at 24 h post-infection but were markedly reduced to 0.47 at 48 h (**Figure 2E**). This delayed response may be attributed to the lower phagocytic capacity of MLE-12 cells and the slower onset of BCG infection. Together, these findings indicate that miR-9-5p expression is consistently suppressed in host cells following mycobacterial infection.

We next assessed whether BCG infection affects miR-9-5p abundance in EVs. Notably, EVs derived from RAW264.7 macrophages at 6 h post-BCG infection showed a dramatic reduction in the relative expression level miR-9-5p, decreasing to 0.2, or approximately 20% of the uninfected control (**Figure 2F**). These results suggest that mycobacterial infection suppresses miR-9-5p expression not only within host cells but also within EV cargo, indicating reduced EV-mediated transfer of this regulatory miRNA under infectious conditions.

### 3.4 miR-9-5p enhances the ability of macrophages to inhibit intracellular mycobacterial growth

**Figure 3:**
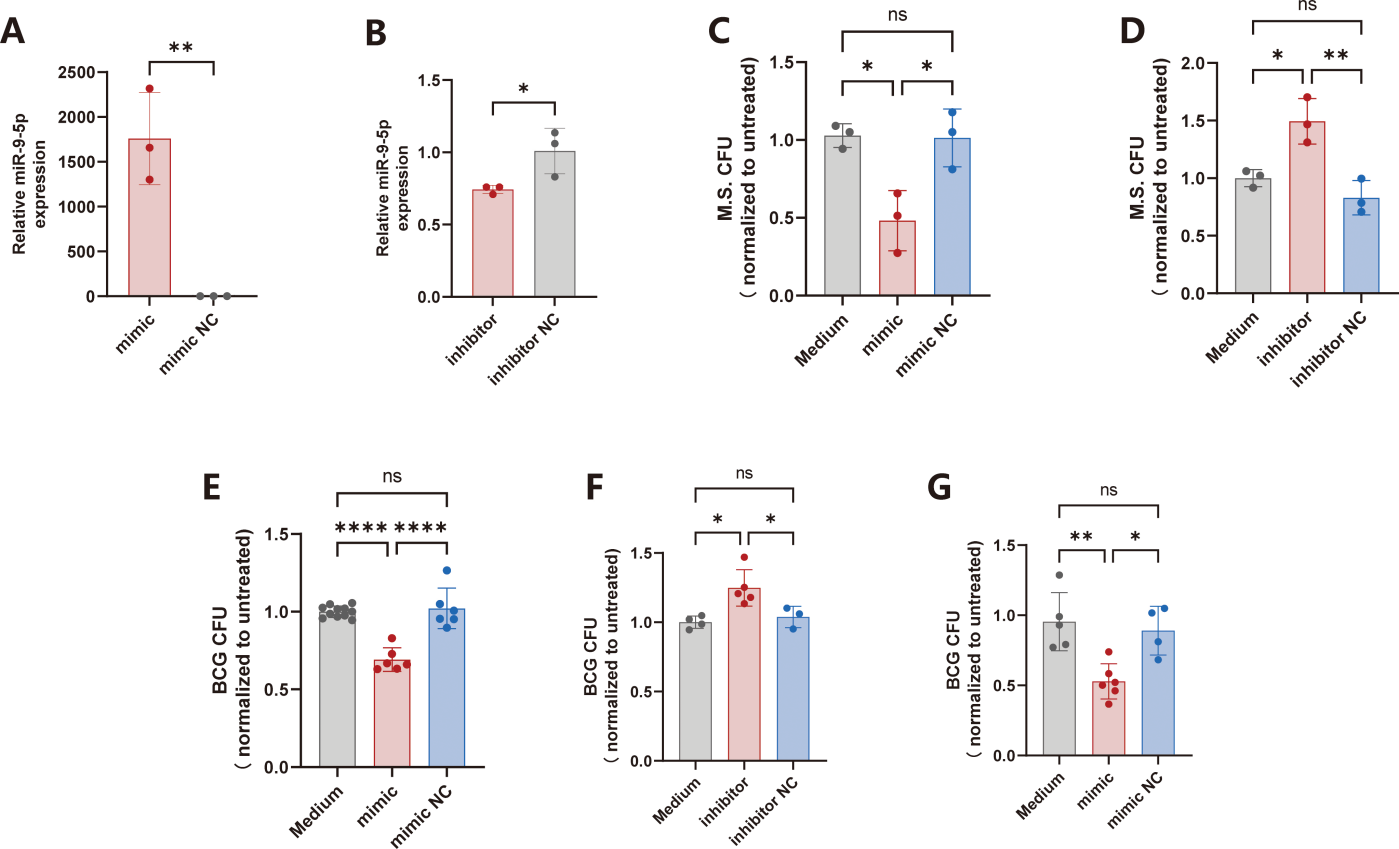
miR-9-5p suppresses the intracellular growth of *M. smegmatis* and BCG in macrophages. **(A)** RAW264.7 cells were transfected with miR-9-5p mimics, and miR-9-5p expression was assessed by RT-qPCR. **(B)** RAW264.7 cells were transfected with miR-9-5p inhibitors, and miR-9-5p expression was determined by RT-qPCR. **(C)** RAW264.7 cells transfected with miR-9-5p mimics or controls were infected with *M. smegmatis*, and CFU were quantified after 12h incubation. **(D)** RAW264.7 cells transfected with miR-9-5p inhibitors or controls were infected with *M. smegmatis*, and CFU were counted after 12h incubation. **(E)** RAW264.7 cells transfected with miR-9-5p mimics or controls were infected with BCG, and BCG CFU were counted after 3-day incubation. **(F)** RAW264.7 cells transfected with miR-9-5p inhibitors or controls were infected with BCG, and BCG CFU were counted after 3-day incubation. **(G)** THP-1 cells transfected with miR-9-5p mimics or controls were infected with BCG, and BCG CFU were counted after 3-day incubation. Results are expressed as mean ± SD. *p < 0.05, **p < 0.01, ****p < 0.0001. ns p > 0.05. Statistical significance was determined using Student’s t-test **(A, B)** or one-way ANOVA **(C–G)**. Data represent 3 independent experiments.

Given the pronounced downregulation of miR-9-5p following mycobacterial infection, we hypothesized that miR-9-5p may enhance the antimycobacterial activity of host macrophages. To test this, RAW264.7 cells were transfected with miR-9-5p mimics or inhibitors, and RT-qPCR confirmed a 1700-fold increase in miR-9-5p expression following mimic transfection (**Figure 3A**), whereas inhibitor transfection markedly reduced endogenous miR-9-5p levels (**Figure 3B**), validating the transfection efficiency.

We next evaluated the functional role of miR-9-5p in macrophage antibacterial activity against fast-growing *M. smegmatis* infection. Overexpression of miR-9-5p significantly reduced the average relative CFU value of *M. smegmatis* to 0.5, representing an approximately 50% decrease relative to the negative control with relative CFU value as 1 (**Figure 3C**). Conversely, inhibition of miR-9-5p increased *M. smegmatis* relative CFU to 1.5, approximately 1.5-fold higher than the control (**Figure 3D**), indicating the depletion of miR-9-5p impairs macrophage bactericidal activity.

A similar pattern was observed in the slow-growing BCG infection model. In RAW264.7 cells, miR-9-5p mimics reduced intracellular BCG burden to 0.6, a 40% decline compared with the control (**Figure 3E**), whereas miR-9-5p inhibitors significantly increased intracellular BCG loads (**Figure 3F**). Importantly, consistent results were obtained in human THP-1 macrophages, where miR-9-5p mimics reduced the average relative BCG CFU value to 0.5, compared with the average relative BCG CFU value as 1 in the control group (**Figure 3G**). These findings indicate that miR-9-5p enhances the antimycobacterial immunity of macrophages.

Combined with the observed infection-induced suppression of miR-9-5p, these data suggest that miR-9-5p functions as a host-protective molecule, and its downregulation may represent a pathogen-driven strategy to evade macrophage immunity.

### 3.5 miR-9-5p–enriched EVs effectively suppress intracellular mycobacterial growth through ROS related pathways

**Figure 4:**
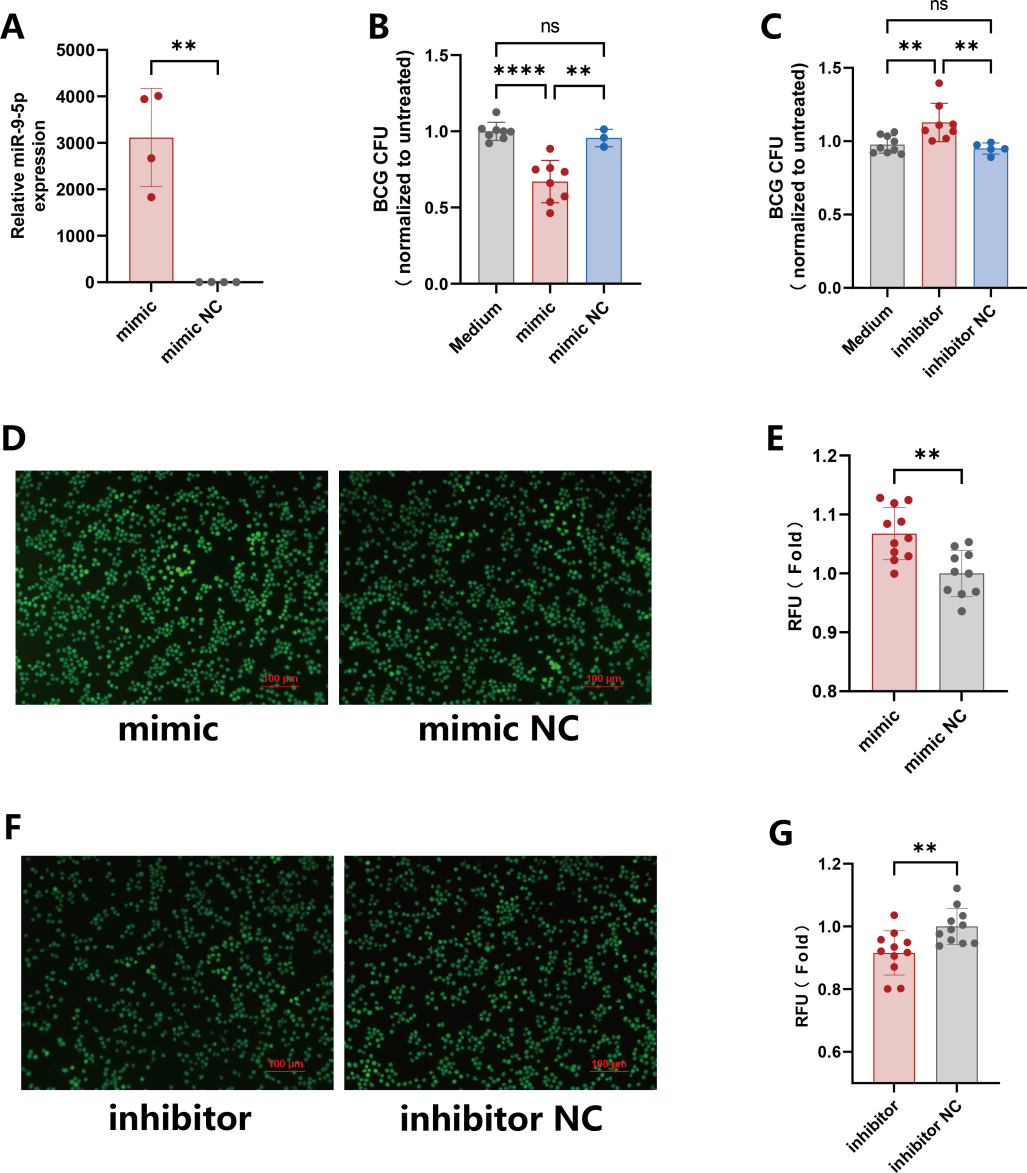
EVs enriched in miR-9-5p enhance antimicrobial activity and promote ROS-mediated anti-mycobacterial responses. **(A)** RAW264.7 cells were transfected with miR-9-5p mimics, and extracellular vesicles were collected 48 h later. Total RNA from EVs was extracted and the enrichment of miR-9-5p was assessed by RT-qPCR. **(B)** EVs derived from miR-9-5p mimics–transfected cells or control EVs were added to BCG-infected RAW264.7 cells. After 3 days of incubation, cells were lysed and BCG CFU were quantified. **(C)** EVs derived from miR-9-5p inhibitors–transfected cells or control EVs were added to BCG-infected RAW264.7 cells, and intracellular BCG CFU were determined. **(D)** THP-1 cells were transfected with miR-9-5p mimics or control, and intracellular ROS levels were visualized by fluorescence microscopy using the DCFH-DA probe, where DCF fluorescence indicates ROS production. **(E)** Quantification of ROS fluorescence intensity in THP-1 cells following miR-9-5p mimics transfection. **(F)** THP-1 cells were transfected with miR-9-5p inhibitors or control, and intracellular ROS levels were visualized by fluorescence microscopy using the DCFH-DA probe, where DCF fluorescence indicates ROS production. **(G)** Quantitative analysis of ROS fluorescence intensity in THP-1 cells following miR-9-5p inhibitors transfection. Results are expressed as mean ± SD. **p < 0.01, ****p < 0.0001. ns p > 0.05. Statistical significance was determined using Student’s t-test(A,E,G) or one-way ANOVA(B,C). Data represent 3 independent experiments.

To determine whether miR-9-5p contributes to enhancing the ability of EVs to inhibit intracellular mycobacterial growth, we increased the abundance of miR-9-5p in EVs by transfecting cells with miR-9-5p mimics to produce miR-9-5p-enriched EVs. RT-qPCR revealed that miR-9-5p levels in EVs increased by ~3000-fold (**Figure 4A**), markedly higher than the increase observed in donor cells (1700-fold) (**Figure 3A**), indicating the successful production of miR-9-5p–enriched EVs. Treatment of BCG-infected RAW264.7 cells with miR-9-5p-enriched EVs significantly reduced the relative BCG CFU values to 0.68, compared with the relative BCG CFU values as 1 in the control group (**Figure 4B**), demonstrating that EV-mediated delivery of miR-9-5p enhances the antimycobacterial ability of macrophages. Conversely, the BCG burden in cells treated with EVs derived from miR-9-5p inhibitor-transfected cells was significantly higher than that of controls (**Figure 4C**), indicating that miR-9-5p deficiency impairs the antimicrobial capacity of EVs.

Given the importance of reactive oxygen species (ROS) in the macrophage-mediated killing of intracellular pathogens, we examined the effects of miR-9-5p on ROS production in THP-1 macrophages. Overexpression of miR-9-5p significantly increased ROS levels compared to the controls (**Figures 4D, E**), whereas miR-9-5p deficiency markedly reduced ROS production (**Figures 4F, G**), demonstrating that miR-9-5p might enhance the ability of macrophages to inhibit intracellular mycobacterial growth by increasing ROS production.

### 3.6 EVs might migrate from gut to lung to increasing antimycobacterial immunity of lung

**Figure 5:**
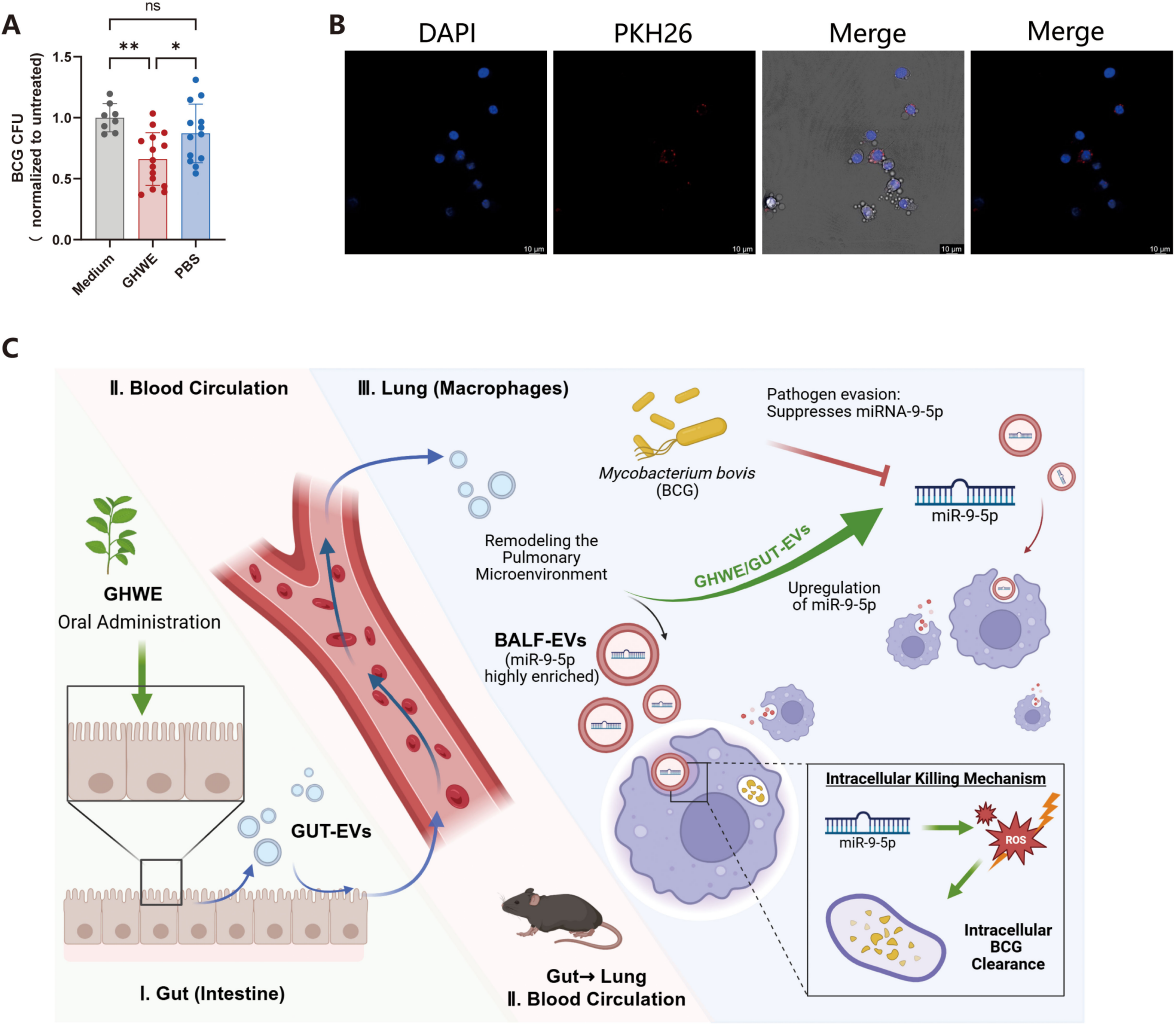
EVs–mediated gut–lung communication enhances host antimycobacterial immunity. **(A)** GUT-EVs (5 μg) derived from PBS- or GHWE-treated mice were added to BCG-infected RAW264.7 cells. After 3 days of incubation, cells were lysed, serially diluted, plated on 7H10 agar, and BCG CFU were quantified. n=15 independent biological replicates per group (each replicate pooled from 8 mice). *p < 0.05, **p < 0.01. ns p > 0.05. **(B)** Confocal microscopy showing red fluorescence signals in RAW264.7 cells, indicating uptake of PKH26-labeled GUT-EVs (scale bar, 10 μm). **(C)** Schematic illustration of the proposed mechanism. GHWE-induced GUT-EVs enter lungs through the systemic circulation and promote the secretion of BALF-EVs enriched in miR-9-5p. The transferred miR-9-5p enhances macrophage antimicrobial activity by promoting intracellular ROS production, thereby suppressing intracellular mycobacterial growth. BALF-EVs, Bronchoalveolar lavage fluid-derived EVs; GUT-EVs, Gut-derived EVs. Created with BioRender.com.

Given that GHWE was administered via oral gavage, it primarily acts in the intestine. To explore how GHWE gavage enhances host immune responses against mycobacterial infection, we isolated GUT-EVs from the intestinal epithelial cells of GHWE-administrated mice using differential ultracentrifugation and examined their antimycobacterial activity of gut-derived EVs. As shown in **Supplementary Figure 1A**, NTA revealed a particle concentration of 8.64 × 10¹¹ particles/mL, with a size distribution predominantly ranging from 160 to 400 nm (**Supplementary Figure 1A**), indicating that the GUT-EV population in this study primarily consists of medium-to-large EVs or microvesicles rather than classical small exosomes. TEM further confirmed the presence of membrane-bound, electron-dense vesicles exhibiting a characteristic cup-shaped morphology(**Supplementary Figure 1B**). Furthermore, we found that GUT-EVs significantly suppressed intracellular mycobacterial growth in macrophages (**Figure 5A**). Additionally, our miRNA sequencing analysis results of GUT-EVs revealed that, unlike in BALF-EVs the expression of miR-9-5p in the GUT-EVs of GHWE-administrated mice was not significantly higher than that in controls (**Supplementary Figures 1C, D**).

To determine the *in vivo* distribution and chemotaxis of GUT-EVs after injection, DiR-labeled EVs were injected intravenously into mice. Whole-body fluorescence imaging at 2 h post-injection (**Supplementary Figure 2A**) and quantitative analysis (**Supplementary Figure 2B**) revealed that fluorescently labeled EVs predominantly accumulated in the liver, indicating systemic transport to metabolic and immune-relevant organs. Time-course imaging at 2 h, 6 h, 24 h, and 48 h showed a gradual decline in fluorescence intensity (**Supplementary Figure 2C**). Ex vivo imaging of major organs (**Supplementary Figure 2D**) demonstrated strong accumulation in the liver, lung, and spleen. Quantitative data indicated that lung fluorescence peaked at 24 h before gradually decreasing (**Supplementary Figures 2E, F**), suggesting efficient delivery and transient enrichment of GUT-EVs in the lung.

To determine whether GUT-EVs can be internalized by macrophages, PKH26-labeled EVs were incubated with RAW264.7 cells. Confocal microscopy showed robust intracellular red fluorescence of EVs (**Figure 5B**), confirming the efficient uptake of GUT-EVs by macrophages. Importantly, when combined with our functional assays demonstrating significantly reduced intracellular BCG burdens, this robust internalization establishes a direct functional link, demonstrating that EV uptake causally dictates the enhanced antimycobacterial capacity of the recipient cells.

These findings collectively indicate that gut-derived EVs could enter the systemic circulation, reach the lung, and interact with pulmonary immune cells-supporting a potential gut–lung axis through which GHWE modulates pulmonary antimycobacterial immunity (**Figure 5C**).

## 4 Discussions

In this study, to investigate the anti-tuberculosis activity of green tea bioactive components, we observed that stimulating PBMCs with GHWE was associated with an enhanced inhibition of intracellular *Mtb* growth. Subsequently, we found that GHWE gavage correlated with a significant reduction in the pulmonary bacterial load of mycobacteria infected mice. Moreover, GHWE administration enhanced the antibacterial activity of EVs derived from both the intestine and the BALF. Through tracking experiments, we demonstrated that intravenously injected GUT-EVs have the capacity to accumulate in the lungs. Taken together, rather than definitively proving a causal pathway, these associative findings strongly suggest that GHWE administration may bolster the host’s distal immune response against tuberculosis infection, highlighting the “gut-lung axis” as a potential regulatory mechanism.

Furthermore, the enhancement of anti-tuberculosis immunity induced by GHWE may involve multiple parallel or intersecting biological pathways. First, green tea is rich in bioactive polyphenols, particularly catechins such as EGCG, epicatechin gallate (ECG), and epi-gallocatechin (EGC), which possess strong antioxidant and immunomodulatory properties (35). Studies indicate that hot-water extraction can significantly enhance the yield of water-soluble active compounds, thereby enriching catechins (36, 37). Elevated concentrations of key components such as EGCG in the extract potentiate their biological activity *in vivo* (38). Increasing evidence suggests that EGCG not only acts intracellularly but can also modulate host immune responses indirectly via EVs. For example, cardiomyocytes pretreated with EGCG release EVs enriched in miR-30a, which can be taken up by neighboring cardiomyocytes to inhibit apoptosis and autophagy, thereby exerting cardioprotective effects (39). These observations imply that the bioactive compounds in GHWE may exert distal immunoregulatory effects through EV-associated pathways or other systemic mechanisms.

In addition, modulation of the gut microbiota and the gut–lung axis may represent another important mechanism underlying the immunological effects of GHWE. Accumulating evidence indicates that green tea can modulate the gut microbiota (40), altering microbial metabolites and influencing distal organ immunity through “gut-organ” axes (41). Short-term green tea supplementation has been shown to rapidly reshape gut microbial composition and improve mucosal metabolism and inflammatory markers (7), suggesting that dietary polyphenols may function as “microbe-host mediators” to exert systemic health effects. GHWE may also act as a prebiotic-like agent by increasing the abundance of beneficial bacteria, such as *Akkermansia muciniphila* or *Lactobacillus (*42, 43), thereby promoting the production of short-chain fatty acids (SCFAs) (44), which have been shown to play important roles in regulating anti-infective immunity in distal organs (45). In the context of tuberculosis, an increased abundance of *Bifidobacterium* has been positively associated with enhanced host resistance to infection (46). *Lactobacillus* species not only possess intrinsic immunomodulatory properties but have also been explored as engineered antigen delivery vehicles to enhance tuberculosis-related immune responses (47). Moreover, *Lactobacillus* supplementation has been reported to attenuate pulmonary inflammation and tissue damage during anti-tuberculosis treatment, highlighting its role in regulating inflammatory cytokine expression and maintaining local immune homeostasis (48). These microbial taxa are therefore considered important contributors to gut homeostasis and to the modulation of the pulmonary immune microenvironment via the gut–lung axis. It should be noted that the present study did not systematically evaluate changes in gut microbiota composition or metabolic profiles following GHWE administration. Future studies integrating multi-omics approaches are warranted to elucidate the roles of microbial metabolites and gut-derived EVs in GHWE-mediated enhancement of anti-tuberculosis immunity, thereby providing a more comprehensive mechanistic framework.

In the present study, both BALF-EVs and GUT-EVs isolated from mice gavaged with GHWE markedly inhibited intracellular mycobacterial growth in macrophages, indicating their potential roles in antimycobacterial immunity. EVs are increasingly recognized as active signaling entities that influence recipient cell behaviors through the transfer of bioactive cargo. Consistent with this paradigm, we confirmed that macrophages efficiently internalize EVs and that EV exposure is sufficient to enhance intracellular mycobacterial clearance. These findings suggest that EV uptake represents a functional step in modulating macrophage antimicrobial responses rather than a passive bystander phenomenon. It should be noted that the present study demonstrated this functional linkage rather than defining a detailed dose–effect relationship between EV abundance and bactericidal activity. Moreover, regarding the mechanistic mediators, the inherent heterogeneity of the isolated EVs poses a significant challenge. The vesicle populations utilized in our study likely represent a diverse mixture of exosomes and microvesicles originating from various cell types, meaning the precise EV subset responsible for the observed antimycobacterial effects remains unidentified. Therefore, future studies incorporating EV dose titration, single-vesicle resolution to address heterogeneity, and cargo-specific manipulation will be necessary to delineate the quantitative and mechanistic aspects of EV-mediated antimycobacterial immunity.

Furthermore, whole-transcriptome sequencing revealed a significant enrichment of miR-9-5p in BALF-EVs; subsequent functional assays demonstrated that miR-9-5p enhances the capacity of macrophages to suppress intracellular mycobacterial proliferation. However, a critical mechanistic question remains: whether the miR-9-5p-enriched EVs observed in the lung are directly transported from the gut or locally produced by pulmonary cells. Our whole-transcriptome sequencing of GUT-EVs revealed that miR-9-5p levels remained stable following GHWE administration, a finding that contrasted with the marked enrichment of miR-9-5p in BALF-EVs. This divergence in miRNA profiles suggests a compartmentalized response rather than direct cargo transport. While our current labeling experiments confirm the physical accessibility of GUT-EVs to the lung, the lack of miR-9-5p enrichment in these vesicles implies that they may not serve as the direct source of this specific miRNA. Instead, it is more plausible that GUT-EVs act as inter-organ signaling mediators that stimulate lung-resident cells—such as alveolar macrophages or epithelial cells—to endogenously upregulate and release miR-9-5p-enriched EVs. Supporting this notion, in multiple mouse models of inflammatory lung diseases, including infection-exacerbated asthma, COPD, and acute lung injury (ALI), EVs present in the airway lining fluid have been shown to carry pro-inflammatory cytokines such as IL-18, IL-1β, and TNF-α, thereby promoting neutrophil recruitment and key pathological features of lung inflammation (49–51). These findings illustrate that EV-mediated signaling within the lung can potently reshape local immune responses. Despite technical limitations in definitively distinguishing the cellular origin of these vesicles *in vivo*, our observation regarding molecular heterogeneity provides a strong indication that the miR-9-5p-mediated effect is primarily a localized pulmonary response triggered by gut-derived signals.

While our findings highlight the antimycobacterial role of miR-9-5p, it is important to acknowledge its pleiotropic and non-specific effects on broader immune functions (52, 53). For instance, it can modulate broad inflammatory signaling cascades (such as AKT/MAPK) and regulate chemokine expression (49). Furthermore, miR-9-5p has the potential to drive macrophage polarization toward a pro-inflammatory M1 phenotype via axes like SIRT1/NF-κB (49), thereby altering the basal activation state of the cells. Beyond macrophages, EV-delivered miR-9-5p might exert systemic immunomodulatory effects across other immune subsets, such as influencing T-cell differentiation in the microenvironment. Therefore, the enhanced *in vivo* anti-tuberculosis efficacy we observed is likely a synergistic outcome of both direct ROS-mediated bactericidal activity and broader, pleiotropic immune network activation.

Although our study demonstrates that GHWE treatment significantly reduces bacterial burden in the lungs of mice, the specific immune cell populations responsible *in vivo* remain to be determined. Based on our *in vitro* experiments, macrophages appear to be the key mediators. During the early stages of *Mtb* aerosol infection, alveolar macrophages (AMs) (54), as the first line of defense, may enhance antibacterial activity by internalizing GUT-EVs or producing BALF-EVs containing miR-9-5p in response to local stimuli. As infection progresses, circulating monocytes are recruited to the lung and differentiate into monocyte−derived macrophages (MDMs), which become the dominant infected macrophage population and may contribute to antimicrobial defense and inflammatory regulation (55). In addition, the involvement of other immune cell types, such as neutrophils or dendritic cells, cannot be excluded in the reduction of the pulmonary bacterial burden. Our mechanistic studies primarily employed the macrophages cell lines RAW264.7 and THP-1, which are widely used to study macrophage function, although they cannot fully recapitulate the functional heterogeneity or spatial distribution of primary macrophages (e.g., AMs or interstitial macrophages) in the lung microenvironment. Therefore, further studies using primary cell isolation or single-cell sequencing will be valuable in identifying the specific cell subsets involved.

It is important to consider that intravenous administration of EVs allows for the rapid assessment of organ distribution but does not fully reflect the physiological migration from the gut to the lung along the gut–lung axis. Thus, the observed lung localization provides preliminary evidence of EV delivery feasibility rather than precise *in vivo* trafficking or targeting specificity. DiR is a lipophilic dye that labels EVs via non-covalent association with lipid membranes. While it is simple, efficient, and applicable to EVs from various sources, DiR can also associate with other membrane structures or form aggregates, and the signal may persist after EV degradation (56). Therefore, DiR-based imaging provides semi-quantitative, short-term information on EV localization and should not be over-interpreted as reflecting the exact physiological migration or targeting of EVs *in vivo*.

To systematically evaluate the immunomodulatory effects of GHWE and GHWE-induced EVs on pulmonary antimycobacterial immunity, we employed well-justified cellular and murine models of acute BCG infection as a “proof-of-concept” study. Previous studies have demonstrated that BCG infection not only elicits adaptive immune responses but also activates trained innate immunity through the epigenetic and metabolic reprogramming of monocytes/macrophages, thereby providing important mechanistic insights into host antimycobacterial defense while offering clear advantages in biosafety and experimental reproducibility (57, 58). Our findings demonstrate that GHWE and its induced EVs significantly modulate the antimycobacterial functions of macrophages and innate immune cell populations, providing a strong experimental rationale for further validation in *Mtb* infection models.

In summary, this study elucidates a novel molecular mechanism by which dietary intervention modulates EV miRNA profiles, specifically highlighting how miR-9-5p-mediated ROS activation enhances host antimycobacterial immunity. These findings provide an important experimental basis and conceptual framework for exploring dietary modulation and EV-based miRNA delivery as strategies to bolster host defense against tuberculosis. Nevertheless, several critical questions remain to be addressed in future research. First, the complex physiological and metabolic differences between mice and humans mean that the bioavailability, systemic distribution, and EV-mediated trafficking of green tea bioactive compounds may vary significantly in clinical settings. Second, dietary components typically exert mild, modulatory effects rather than potent, acute pharmacological actions. Therefore, we emphasize that interventions like GHWE should be viewed strictly as potential adjunctive or host-directed supportive strategies, rather than standalone therapeutic alternatives to standard anti-tuberculosis regimens. Although the precise systemic mechanisms and full translational potential remain to be elucidated, our results underscore the promise of targeting cross-organ communication networks. Future studies should prioritize validating these findings in well-controlled human population cohorts and exploring the clinical feasibility of EV-mediated or dietary interventions as adjunctive immunotherapy for tuberculosis.

## Data Availability

The datasets presented in this study can be found in online repositories. The names of the repository/repositories and accession number(s) can be found in the article/**Supplementary Material**.

## Supplementary material

Supplementary Figure 1 Characterization and miRNA transcriptomic profiling of GUT-EVs following GHWE administration. **(A)** NTA showing the size distribution and concentration of GUT-EVs. **(B)** TEM image of GUT-EVs, showing the characteristic cup-shaped vesicular morphology (scale bar, 500 nm). **(C)** miRNA sequencing of GUT-EVs followed by principal component analysis (PCA). **(D)** Volcano plot illustrating the differentially expressed miRNAs in GUT-EVs between the PBS and GHWE groups. Red and green dots represent significantly up-regulated and down-regulated miRNAs, respectively.

Supplementary Figure 2 GUT-EVs reach the lung via systemic circulation and regulate pulmonary immune responses. **(A)** DiR-labeled GUT-EVs were injected intravenously into wild-type mice, followed by whole-body fluorescence imaging at 2 h post-injection and quantitative analysis shown in **(B)**. **(C)** Whole-body fluorescence imaging at 2 h, 6 h, 24 h, and 48 h after intravenous injection of DiR-labeled GUT-EVs. **(D)** Mice were euthanized at 2 h, 6 h, 24 h, and 48 h following EVs administration, and major organs (heart, lung, liver, stomach, intestine, spleen, and kidney) were collected for ex vivo fluorescence imaging. Quantification is shown in **(E)**. **(F)** Quantitative analysis of fluorescence intensity in lung tissues across groups.
