# Supplementary figures and images for: Enhancement of host anti-mycobacterial immunity by green tea hot-water extract is mediated through miR-9-5p-enriched lung extracellular vesicles
Source: Front Immunol. 2026 Apr 1;17:1786162. doi: 10.3389/fimmu.2026.1786162 (PMC13079060; doi:10.3389/fimmu.2026.1786162)

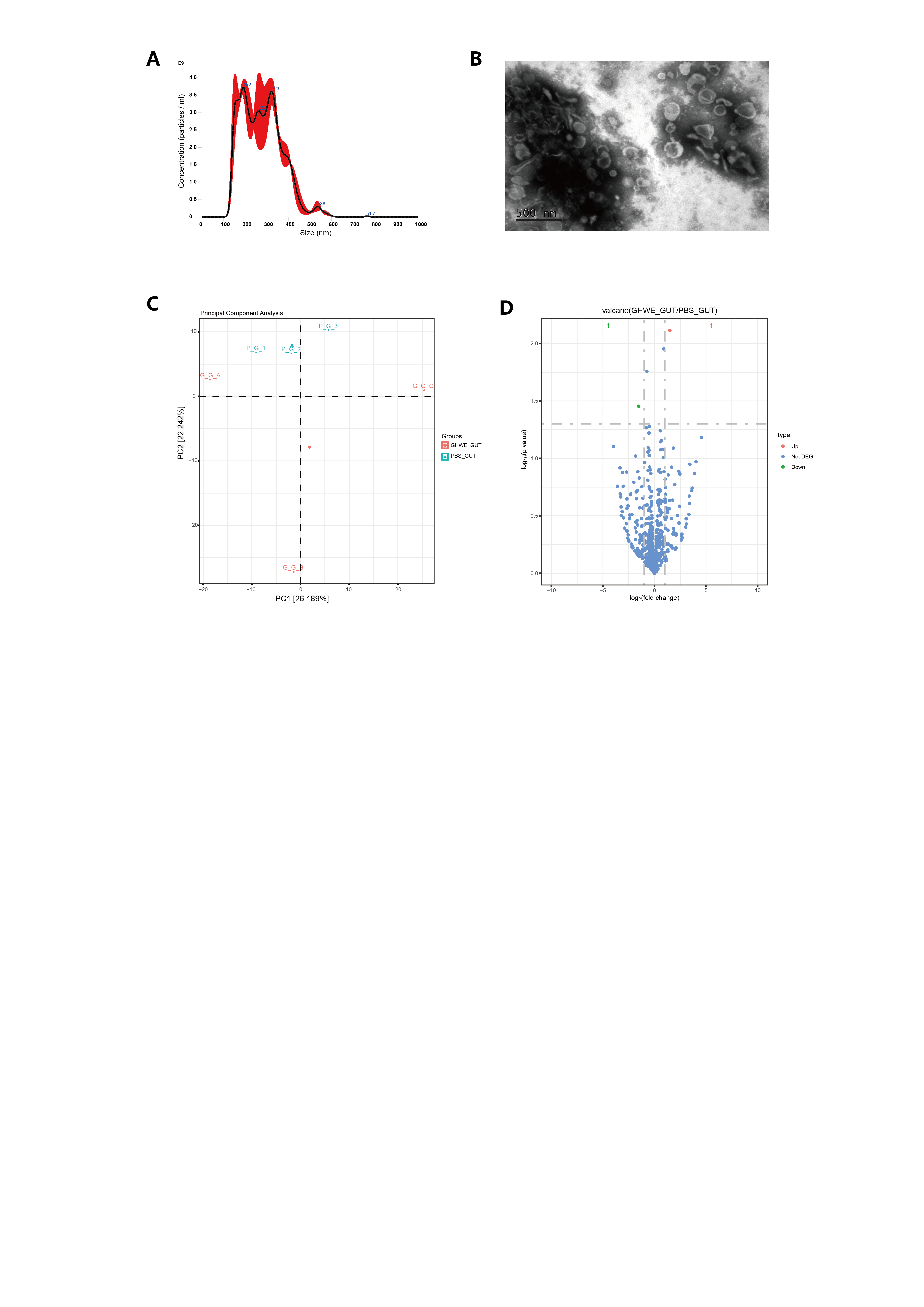

Supplement: Supplementary Figure 1 — Characterization and miRNA transcriptomic profiling of GUT-EVs following GHWE administration. (A) NTA showing the size distribution and concentration of GUT-EVs. (B) TEM image of GUT-EVs, showing the characteristic cup-shaped vesicular morphology (scale bar, 500 nm). (C) miRNA sequencing of GUT-EVs followed by principal component analysis (PCA). (D) Volcano plot illustrating the differentially expressed miRNAs in GUT-EVs between the PBS and GHWE groups. Red and green dots represent significantly up-regulated and down-regulated miRNAs, respectively. [file Image1.png]

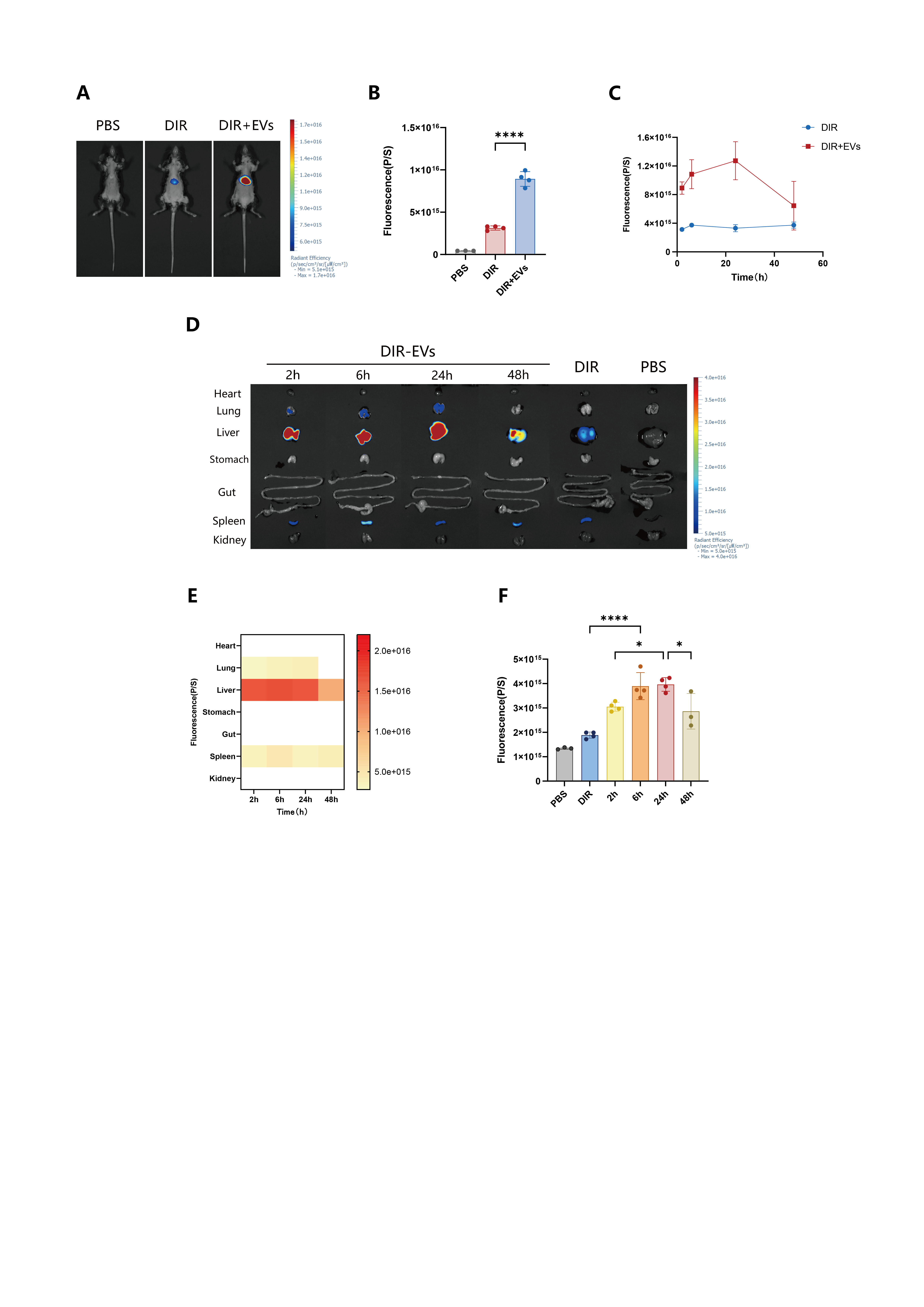

Supplement: Supplementary Figure 2 — GUT-EVs reach the lung via systemic circulation and regulate pulmonary immune responses. (A) DiR-labeled GUT-EVs were injected intravenously into wild-type mice, followed by whole-body fluorescence imaging at 2 h post-injection and quantitative analysis shown in (B). (C) Whole-body fluorescence imaging at 2 h, 6 h, 24 h, and 48 h after intravenous injection of DiR-labeled GUT-EVs. (D) Mice were euthanized at 2 h, 6 h, 24 h, and 48 h following EVs administration, and major organs (heart, lung, liver, stomach, intestine, spleen, and kidney) were collected for ex vivo fluorescence imaging. Quantification is shown in (E). (F) Quantitative analysis of fluorescence intensity in lung tissues across groups. [file Image2.png]
